# Hierarchical Reasoning Gates for Multimodal Large Language Models in Dental Image Interpretation: A Prompt-Level Protocol for Improving Accuracy

**DOI:** 10.3390/jimaging12100508

**Published:** 2026-10-13

**Authors:** Márcio De Menezes, Odirlei Arruda Malaspina, Guillaume Anthony Odri, Cristiano Pires e Silva, Raphael Olszewski

**Affiliations:** 1 School of Health Science, State University of Amazonas, Manaus 69065-001, Brazil; mamenezes@uea.edu.br (M.D.M.); omalaspina@uea.edu.br (O.A.M.); cpsilva@uea.edu.br (C.P.e.S.); 2 Service de Chirurgie Orthopédique et Traumatologique, Centre Hospitalier Universitaire d’Orléans, 45100 Orléans, France; guillaume-anthony.odri@chu-orleans.fr; 3 Innovative Therapies and Nanomedicine (InTheRNA), Inserm U1364, University of Orléans, 45100 Orléans, France; 4 Faculty of Medicine, Lazarski University, 02-662 Warszawa, Poland; 5 Université Catholique de Louvain, 1348 Louvain-La-Neuve, Belgium; 6 Department of Surgical Dentistry and Maxillofacial Surgery, Danylo Halytsky Lviv National Medical University, 79010 Lviv, Ukraine

**Keywords:** artificial intelligence, image interpretation, multimodal large language models, prompt engineering, Hierarchical Reasoning Gates, dental radiography

## Abstract

Multimodal large language models (MLLMs) have been used to support dental image interpretation, but performance may depend on prompt structure. We proposed Hierarchical Reasoning Gates (HRG), a prompt-level protocol organizing image interpretation into sequential stages with validation before progression. Forty anonymized panoramic radiographs of impacted mandibular molars were analyzed using three prompt architectures: P1, zero-shot natural language; P2, a five-step structured workflow followed by a final internal-validation checkpoint; and P3, HRG with 10 macro-stages and 35 procedural control blocks. Outputs were compared with expert radiology reference reports using five predefined variables. Mean Total Score was 3.52 ± 0.85, 3.58 ± 0.71, and 4.35 ± 0.74 for P1, P2, and P3, respectively; Full Accuracy was 10.0%, 5.0%, and 50.0%. Reports with errors in three or more variables occurred in 12.5%, 7.5%, and 0%, respectively. Only Apparent Anatomical Relationship differed significantly across prompts after Holm adjustment. Test–retest repeatability was high for several variables but lower for Apparent Anatomical Relationship in P3. HRG was associated with higher image-interpretation performance and fewer complex error patterns, although higher performance did not uniformly translate into greater repeatability. These findings support gated prompt design for controlling dependent steps in MLLM-based image analysis, while clinical use still requires expert supervision.

## 1. Introduction

Multimodal Large Language Models (MLLMs) combine the capability to process and interpret visual information with the ability to understand natural language [1,2,3,4,5]. In multimodal tasks involving multiple related judgments, accurate outputs depend on the consistent integration of visual and textual evidence [2,6]. Task decomposition and verification mechanisms can help coordinate the intermediate decisions required in these complex tasks [7,8].

Natural language prompts can usually produce the desired output, but they do not necessarily control the order of analytical steps. Such instructions may be insufficient for complex problem-solving tasks because natural language can be ambiguous and models are sensitive to small variations, which may lead to semantic shortcuts, logical errors, and hallucinations [9,10,11,12,13]. Prompt design can influence how an MLLM approaches an image. Structured prompts, such as Chain-of-Thought (CoT), analogical prompting, and Tree-of-Thought, can divide a task into sections and encourage the use of intermediate reasoning steps [8], but these instructions do not necessarily enforce logical precedence between dependent inference stages, and longer reasoning chains tend to increase hallucination rates [5,7].

Different studies of artificial intelligence (AI) systems have examined prompt-based interaction, reasoning decomposition, and output consistency. Schellaert et al. [1] argue that large prompt-driven multimodal models should be evaluated as human-centred cognitive aids, since their performance depends on the interaction among the task, the user’s prompting choices, and the system’s responses. Plaat et al. [7] propose that large language models (LLMs) can become agents able to decompose complex objectives into smaller steps. Qasim et al. [14] introduced the Recursive Decomposition of Logical Thoughts (RDoLT), a prompting framework that organizes reasoning into hierarchical steps and evaluates possible responses using multi-feature scoring. These studies indicate that prompt structure, decomposition strategy, and verification mechanisms can affect model performance. In reasoning chains, early failures in perception or interpretation may affect subsequent conclusions and allow inaccuracies to propagate through later stages [7,14].

Hierarchical and staged approaches have shown potential to improve MLLM reasoning by decomposing perception and inference into staged steps, separating recognition from reasoning, and preserving dependencies between low-level evidence and higher-level conclusions [8,15,16,17]. However, the effectiveness of these approaches depends on the structure of the reasoning task and the difficulty of the benchmark datasets used [8,18].

Anatomical image interpretation provides a useful benchmark setting for examining hierarchical prompting because it requires the coordination of perceptual recognition, spatial reasoning, symbolic labeling, and domain-specific constraints, a combination that exposes failure modes not captured by standard language benchmarks [18,19]. Although the literature has not yet established a mature hierarchical prompting framework for dental imaging, this domain can provide a controlled and challenging setting for evaluating whether intermediate validation gates can reduce errors across dependent analytical steps.

We examine whether the use of validation gates to structure dependencies among intermediate decisions reduces the incidence of co-occurring and multicomponent errors in a controlled visual-symbolic task. The contribution is therefore methodological: a prompt-level experimental framework for studying dependency control in multimodal inference. The methodology may provide a reproducible approach for investigating other anatomical interpretation tasks that require consistency across dependent analytical stages and for assessing how control structures may influence co-occurring error patterns and output reliability in MLLMs.

## 2. Materials and Methods

### 2.1. Experimental Design

A benchmark dataset of anonymized dental panoramic images was used to compare three progressively developed prompt architectures. The architectures ranged from basic natural-language instructions to structured hierarchical prompts incorporating validation gates. Each image was assessed using all three prompt architectures, allowing within-image comparisons. The output generated for each radiograph was compared with an expert radiologist report, which served as the image-specific reference standard. The central hypothesis was that a hierarchical prompting framework incorporating validation gates would improve output image-interpretation performance.

### 2.2. Ethical Considerations

The study followed the principles of the Declaration of Helsinki. Ethical approval was granted for the retrospective analysis of pre-existing anonymized data. All images were obtained from the Dental Polyclinic of the State University of Amazonas (UEA) imaging database, Manaus, Brazil, and were anonymized prior to analysis. The study was covered by the approval granted by the State University of Amazonas (UEA) Ethics Committee (CAAE: 89427725.9.0000.5016, approved on 18 June 2025—Field of Study: Health Sciences/Dental Radiology/Artificial Intelligence). No new data collection or patient contact was involved. The radiographs were anonymized rather than pseudonymized, and no personally identifiable information or burned-in patient identifiers was present in the images or associated metadata. To preserve data confidentiality, before image submission, the “Improve the model for everyone” setting was disabled for the account. According to the provider’s data-control documentation, new conversations generated while this setting is disabled are not used to improve the provider’s models. The data were processed in accordance with institutional procedures and Brazilian Law No. 13,709/2018, the Brazilian General Data Protection Law (LGPD).

### 2.3. Benchmark Dataset and Reference Standard

**Dataset.** The experiment used 40 anonymized panoramic radiographs obtained from the UEA institutional repository. The images were obtained from an internal clinical repository rather than from a named public dataset and are not distributed under a public license. Images were included when they showed an impacted mandibular molar requiring assessment of tooth identity, position, orientation, and relationships with adjacent anatomical structures. Images were excluded when image quality was inadequate for interpretation, distortion involved the mandibular molar region, or the region of interest was not fully visualized. No attempt was made to balance the dataset according to Lateralization, Tooth Number, Tooth Angulation, Eruption Status, or Apparent Anatomical Relationship. The benchmark retained the naturally occurring case-level anatomical variability present in the eligible institutional examinations. Consequently, the distribution of radiographic characteristics reflected the biological heterogeneity of the included cases rather than an experimentally imposed sampling pattern.

**Region of Interest Definition.** For each panoramic radiograph, a rectangular region of interest (ROI) was manually delineated using a dashed outline in Microsoft Paint for Windows. The ROI was superimposed on the full panoramic radiograph; the image was not cropped, and the full dental arch remained visible to the model. The ROI limits were adjusted to accommodate anatomical variation across cases and to indicate the impacted mandibular molar and adjacent structures selected for detailed analysis. The annotated images had dimensions of 2616 × 1512 pixels, with a horizontal and vertical resolution of 96 dpi and a 24-bit color depth. The annotated image was saved as a Portable Network Graphics (PNG) file and submitted to the MLLM without additional enhancement, augmentation, or automated preprocessing. The same annotated panoramic image was used across all three prompt architectures. ROI delineation, image preparation, and randomization were performed by the researcher responsible for preparing the image set before radiologist evaluation.

**Reference Standard (RS).** The anonymized images were submitted to an experienced oral and maxillofacial radiologist, who independently evaluated all 40 panoramic radiographs and prepared a reference report for each examination. The assessment considered the five study variables: Tooth Number, Lateralization, Tooth Angulation, Eruption Status, and Apparent Anatomical Relationship. These reports constituted the reference standard for the subsequent evaluation of the AI-generated outputs.

### 2.4. Prompt Architecture and Evaluation Framework

**Figure 1:**
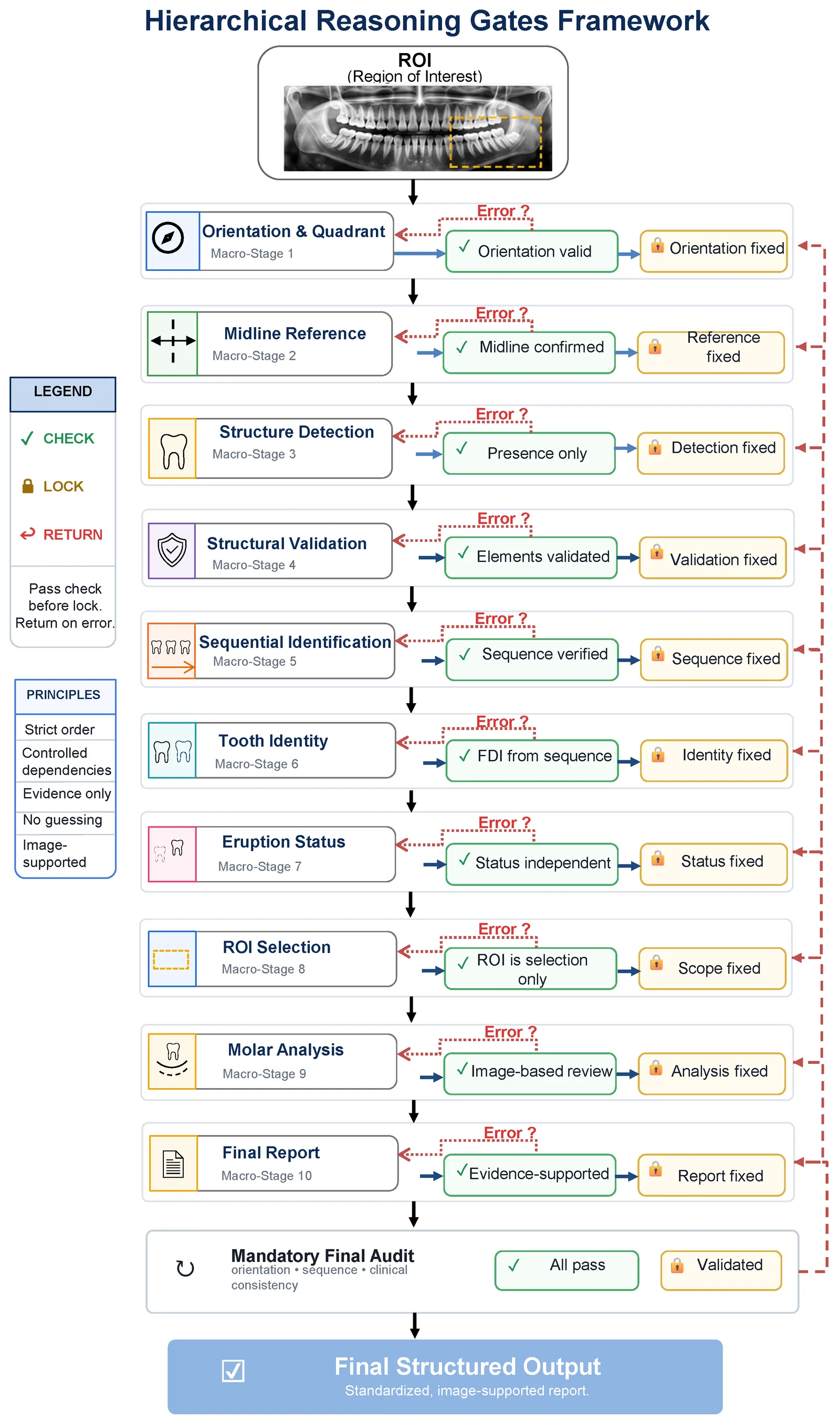
Architecture of the Hierarchical Reasoning Gates (HRG) Framework. Colors and shapes are used for visual organization, while arrows indicate the direction of the reasoning and control flow.

The study followed a two-phase design:

1. Exploratory phase: An experimental set of five panoramic radiographs (excluded from the final benchmark) was used to identify recurrent MLLM errors and to determine which interpretation components could be consistently assessed from the panoramic ROI and compared with corresponding information available in the clinical reports. Five components were retained for the comparative evaluation: Tooth Number, Lateralization, Tooth Angulation, Eruption Status, and Apparent Anatomical Relationship. Recurrent errors identified during this phase, including incorrect image orientation and subsequent errors in tooth identification, also guided the design of the three prompt architectures:
  - Prompt 1 (P1)—baseline: A zero-shot, unstructured natural-language prompt directing the MLLM to act as a radiologist to produce a formal radiographic report without specifying an analytical sequence or validation procedure. It relies on standard descriptive instructions to identify teeth, describe position, and assess anatomical relationships, using general guidelines to avoid assumptions and classify non-visible features as “not assessable”.
  - Prompt 2 (P2)—structured: A systematic architecture that divides the image-interpretation workflow into five sequential steps, followed by a final and mandatory ‘Internal Validation’ checkpoint to verify consistency and eliminate unsupported assumptions before generating a structured report in a predefined format.
  - Prompt 3 (P3)—Hierarchical Reasoning Gates (HRG): The prompt follows a hierarchical, sequential, and auditable workflow built around 10 macro-stages. The framework includes 35 explicitly identified procedural control blocks, comprising gates, checkpoints, tests, locks, and audits. Gates define the conditions for moving forward. Checks confirm that the required criteria have been verified. Locks instruct the model not to proceed before the required validation criteria are addressed and not to revise previously validated decisions. If the final audit identifies an inconsistency, the result is considered invalid, and the process returns to the relevant stage for correction. Figure 1 provides a conceptual overview of HRG architecture.
  The complete operational structure and verbatim wording of P1, P2 and P3 are provided in Supplementary Material S1.
2. Prompt Application and Evaluation Phase: AI-generated outputs were evaluated using five image-interpretation variables: Tooth Number, Lateralization, Tooth Angulation, Eruption Status, and Apparent Anatomical Relationship. Each variable was assessed independently by comparison with the corresponding reference standard. An independent researcher conducted the experiment through the AI interface and was responsible only for collecting, recording, coding, and storing the outputs generated for each image. After AI data collection, the three AI-generated reports for each image were provided to the radiologist without prompt labels. Each AI-generated report was compared with the corresponding reference report, and agreement for each of the five variables was recorded according to the binary scoring criteria described in Section 2.6.

**Figure 2:**
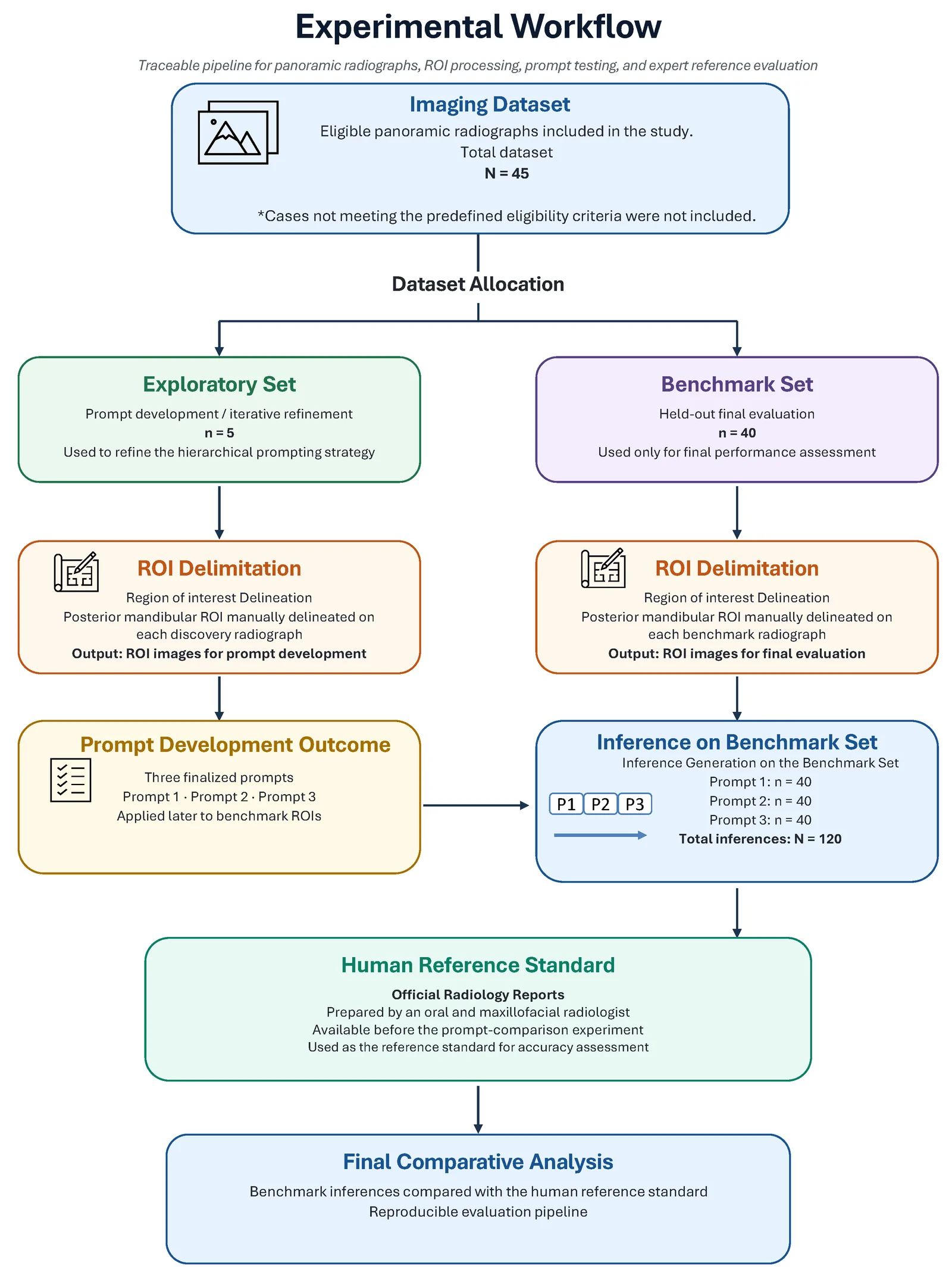
Experimental workflow for the primary prompt-comparison analysis, showing dataset allocation, ROI preparation, assignment of prompt conditions, model inference, and comparison with the expert reports. Colors and shapes are used for visual organization, while arrows indicate the direction of the experimental workflow.

A schematic overview of the primary prompt-comparison workflow, including dataset allocation, ROI processing, inference generation, and expert-based reference reports, is provided in Figure 2.

### 2.5. Experimental Protocol and MLLM Configuration

Data collection was conducted in September 2026.

**Conversational User Interface (CUI) and Agent Configuration.** All multimodal model inferences were performed through the ChatGPT web interface (https://chatgpt.com, accessed on 24 August 2026) using the model option displayed as “GPT-5.6 High” at the time of data collection. Three separate Custom GPT configurations were created, one for each prompt architecture: P1, P2, and P3. For each Custom GPT, the corresponding prompt architecture was uploaded as a plain-text (.txt) Knowledge file rather than entered in the Instructions field. This approach was used to avoid character-length constraints associated with the longer prompt texts. The Custom GPT is a specialized version of ChatGPT that can be configured to perform a defined task. Every agent was assigned only one prompt architecture, and no agent was allowed to access the prompts assigned to the other experimental conditions. The same image and fixed task command were submitted for each case within each condition. The complete verbatim versions of the three prompt architectures and the fixed task command used for every inference are provided in Supplementary Material S1.

**Inference Conditions.** A new chat session was opened for each image with each AI agent (prompt condition). ChatGPT memory was disabled to limit the influence of information from previous cases. Web browsing, external retrieval, and additional tools were disabled during inference. The multimodal assistant generated a radiographic report according to the prompt used.

**Parameter Availability.** The experiment was conducted through the ChatGPT web interface. Standard sampling parameters, including temperature, top-p, random seed, and token limits, were not exposed to the user and could not be manually fixed. Detailed internal model-version information and provider-side computational infrastructure were also unavailable through the interface. All inferences were performed using the same account, Windows operating system, Google Chrome browser, and model option displayed in the interface. The order of prompt application was randomized independently for each image before inference. The six possible permutations of P1, P2, and P3 (P1–P2–P3, P1–P3–P2, P2–P1–P3, P2–P3–P1, P3–P1–P2, and P3–P2–P1) were numbered from 1 to 6. For each of the 40 images, one integer from 1 to 6 was randomly generated in Microsoft Excel using the RANDBETWEEN(1,6) function, and the corresponding permutation determined the order in which the three prompt conditions were applied.

**Data-Control Settings.** The data-control setting permitting submitted content to be used for service improvement was disabled before the images were uploaded. This setting prevented the voluntary contribution of the submitted content for service improvement.

### 2.6. Evaluation Criteria and Scoring Method

Prompt output performance was assessed using five image-interpretation variables: Tooth Number, Lateralization, Tooth Angulation, Eruption Status, and Apparent Anatomical Relationship.

- Binary Scoring Rubric. Each variable was scored independently; an incorrect classification for one variable did not determine the score assigned to the remaining variables. For each variable, a score of 1 was assigned when the model-generated output agreed with the corresponding reference report and 0 when it disagreed or when the information was absent, ambiguous, or internally inconsistent. Tooth Number was recorded according to the Fédération Dentaire Internationale (FDI) two-digit notation. Lateralization referred to the mandibular side. Tooth Angulation was classified according to Winter’s classification. Eruption Status was classified radiographically as erupted, partially erupted, or unerupted. Impaction and eruption status were assessed as distinct radiographic features and were not treated as synonymous. For scoring this variable, the model was required to correctly identify both the eruption status and the presence or absence of impaction as determined by the reference-standard radiologist. Correct identification of only one component was insufficient. Because the model received only the panoramic radiograph, the reference-standard assessment was likewise based exclusively on the panoramic image, without clinical or additional imaging information. Apparent Anatomical Relationship referred to the relationship of the impacted molar with the mandibular canal and adjacent structures as assessable from the two-dimensional panoramic ROI. For this variable, agreement included an apparent proximity to the mandibular canal, no apparent proximity, or recognition that the two-dimensional panoramic image was insufficient for reliable assessment and that three-dimensional evaluation with cone-beam computed tomography (CBCT) would be required.
- The Total Score: Each of the five image-interpretation variables was scored as 0 for an incorrect response or 1 for a correct response. The Total Score was calculated as the sum of these scores, ranging from 0 to 5.
- Full Accuracy (Error-free): A binary outcome coded as 1 when all five variables were correctly identified in the same report and 0 otherwise.
- Co-occurring Error Pattern Analysis: Incorrect outputs were classified according to whether errors affected a single or multiple variables within the same model-generated report. These patterns were used to characterize the frequency and complexity of co-occurring errors across prompt conditions. Because the model’s internal reasoning process was not directly observed, this analysis does not establish causal propagation of an error from one analytical stage to another.
- Primary Output. All model-generated outputs were archived immediately after inference. The primary performance dataset comprised 120 outputs: 40 images evaluated with three prompt architectures. The outputs were used without modification during scoring.
- AI test–retest repeatability. For the test–retest analysis, a second independent set of 120 outputs was obtained using the same experimental protocol, with a newly randomized prompt sequence generated according to the procedure described above. AI test–retest repeatability was assessed by comparing the textual structured image-interpretation decisions produced in two independent runs, resulting in 40 paired Run 1–Run 2 observations for each prompt condition. Free-text outputs from Run 1 and Run 2 were mapped to predefined categories using a coding dictionary established before the comparison. The dictionary defined the allowable categories and semantic equivalences for each variable, so different expressions conveying the same clinical meaning were assigned to the same category. For example, terms such as mesially inclined and mesioangulated were both coded as Mesioangular, whereas Mesioangular and Horizontal were considered distinct categories. For Apparent Anatomical Relationship, outputs were classified as Not evaluable, Close/close contact, Distant/separated, or Mixed. Thus, expressions such as proximity and intimate relationship with the canal were considered equivalent and coded as Close/close contact, whereas statements indicating bony separation were coded as Distant/separated. Lateralization was determined from the FDI tooth identity or quadrant stated in the output rather than from the visual side of the ROI. The five study variables—Tooth Number, Lateralization, Tooth Angulation, Eruption Status, and Apparent Anatomical Relationship—were evaluated independently. Two outputs were considered concordant when the same predefined category was assigned to the corresponding variable in both runs. Repeatability was assessed separately for P1, P2, and P3 using all 40 cases.
- Inter-rater agreement. Inter-rater agreement was assessed across the complete set of 120 image–prompt evaluations (40 radiographs evaluated with P1, P2, and P3) by two independent dental practitioners. They independently evaluated the AI-generated responses and were blinded to both the reference-standard reports and each other’s classifications. For each image–prompt evaluation, they independently classified the five study variables as correct (1) or incorrect (0) based on the predefined scoring criteria and their assessment of the corresponding radiographic image. Their classifications were subsequently compared with each other and with the classifications derived from the reference standard.
  These ratings were obtained exclusively for a separate inter-rater agreement analysis and were not used to determine or modify the primary study outcomes. No consensus or adjudication was performed; disagreements were intentionally retained because the purpose of this analysis was to quantify agreement between independent evaluations. Primary study scoring remained based on comparison with the reference standard established by the oral and maxillofacial radiologist.
- Reproducibility. The experimental protocol was designed and reported to facilitate methodological reproducibility. The study describes the experimental design, model condition, prompt-architecture logic, ROI procedure, predefined image-interpretation components, scoring criteria, and statistical analysis. The experimental procedure was standardized across the three prompt conditions. Each image was assessed separately with P1, P2, and P3, using an independent session for every assessment and identical evaluation criteria throughout. The complete prompts for P1, P2, and P3 are provided in the Supplementary Material. These methodological elements allow the experimental workflow to be replicated using other eligible panoramic radiographs and corresponding expert reference reports. Therefore, reproducibility in the present study primarily concerns replication of the experimental protocol and analytical procedure.

### 2.7. Statistical Analysis

Descriptive statistics were used to summarize overall performance. Total Score was compared across prompt architectures using the Friedman test, with Kendall’s W reported as the effect-size measure. Prespecified pairwise two-sided exact Wilcoxon signed-rank comparisons were to be performed only if the Friedman omnibus test reached *p* < 0.05, with Holm adjustment applied to the resulting pairwise *p*-values. For Wilcoxon comparisons, zero differences were discarded, tied absolute differences were assigned average ranks, and two-sided exact conditional *p*-values were calculated without continuity correction. Full Accuracy was analyzed using Cochran’s Q test; prespecified pairwise exact McNemar comparisons were to be performed only if the corresponding omnibus test reached *p* < 0.05. For the five prespecified binary image-interpretation variables, Cochran’s Q tests were performed where estimable, and Holm adjustment was applied across the prespecified five-variable family. Pairwise exact McNemar comparisons were performed only for variables whose Holm-adjusted omnibus *p*-value was <0.05. Holm adjustment was applied separately within each three-comparison pairwise family for Total Score and Full Accuracy. For the five image-interpretation variables, the five omnibus *p*-values constituted a single Holm-adjusted family; when a variable passed this omnibus gate, its three pairwise McNemar *p*-values were Holm-adjusted within that variable. A significance level of 5% was adopted.

For AI test–retest repeatability, observed agreement between Run 1 and Run 2 was calculated for each structured variable, with 95% confidence intervals estimated using the two-sided Wilson score method. Gwet’s Agreement Coefficient 1 (AC1) with paired-bootstrap 95% confidence intervals and Cohen’s κ were used as chance-corrected agreement coefficients.

Inter-rater agreement was summarized using pairwise observed agreement. Overall chance-corrected agreement among the two independent raters and the reference specialist was assessed using Fleiss’ κ and Gwet’s AC1. Statistical analyses were performed in R version 4.4.3 (R Foundation for Statistical Computing, Vienna, Austria).

## 3. Results

**Figure 3:**
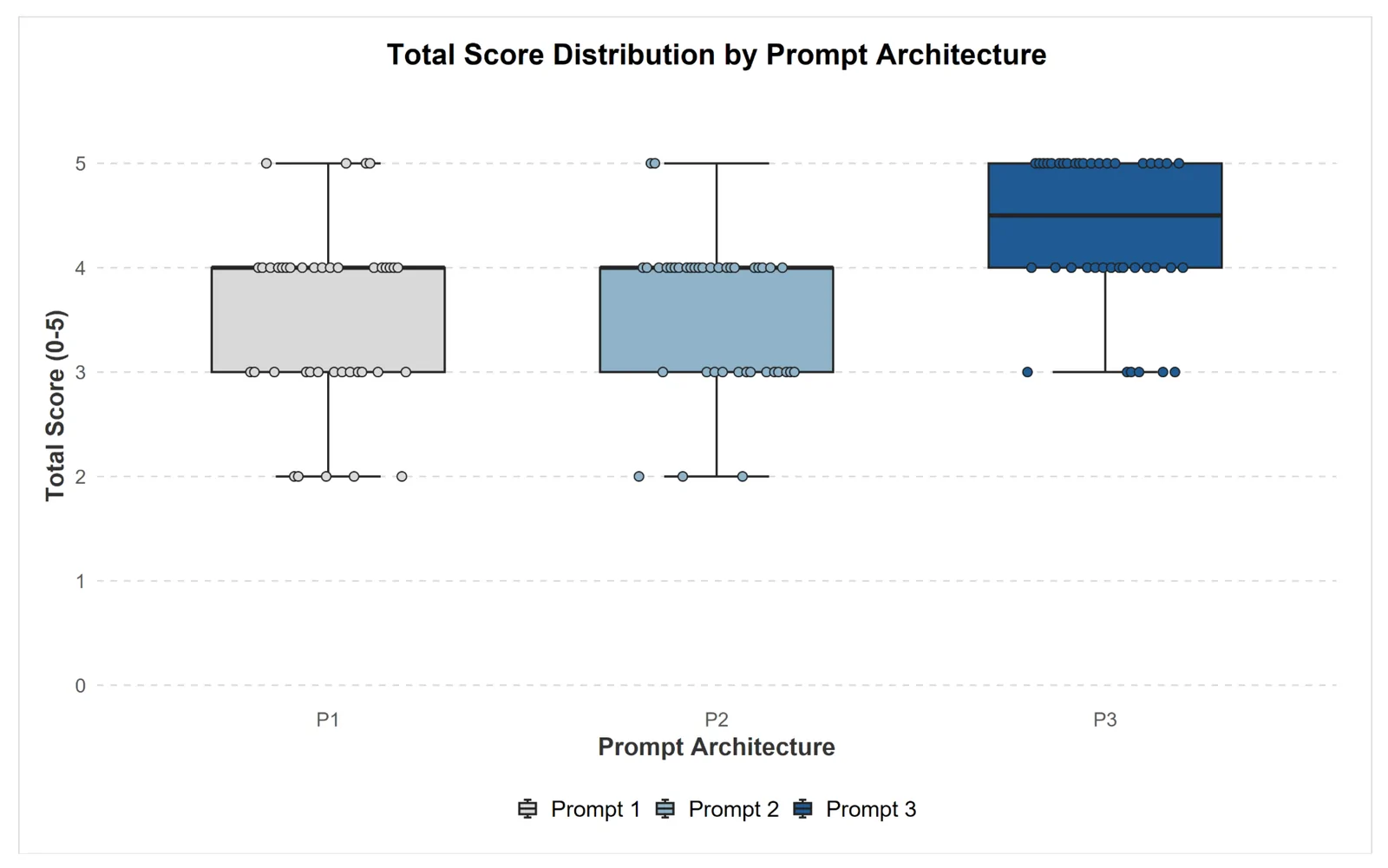
Distribution of Total Scores across prompt architectures. Dots represent individual case-level observations.

One hundred and twenty model-generated reports were obtained from 40 panoramic radiographs, with each image evaluated using all three prompt architectures. Among the 40 panoramic radiographs, 7 cases (17.5%) were classified as erupted, 10 (25.0%) as partially erupted, and 23 (57.5%) as unerupted according to the radiographic reference standard. No case was classified as non-assessable. Therefore, the accuracy of a naïve strategy assigning the majority category (unerupted) to all cases would be 57.5%. Descriptive statistics for Total Score are presented in Table 1. Mean Total Score was 3.52 ± 0.85 for P1, 3.58 ± 0.71 for P2, and 4.35 ± 0.74 for P3. The median score was 4.0 for P1 and P2 and 4.5 for P3. The interquartile range was 3.0–4.0 for both P1 and P2 and 4.0–5.0 for P3. Scores ranged from 2 to 5 for P1 and P2 and from 3 to 5 for P3. The distribution of Total Scores across prompt architectures is shown in Figure 3.

Paired repeated-measures analyses were performed to determine whether Total Score and Full Accuracy differed significantly across prompt architectures (Table 2). Full Accuracy was defined as an error-free report in which all five image-interpretation variables were correctly identified. Total Score differed significantly among prompts according to the Friedman test ($\chi^{2}$(2) = 36.873, *p* < 0.001). The corresponding effect size was Kendall’s W = 0.461. Holm-adjusted pairwise Wilcoxon signed-rank tests showed significantly higher Total Scores for P3 than for P1 (*p* < 0.001) and P2 (*p* < 0.001), whereas no significant difference was observed between P1 and P2 (*p* = 0.817). Full Accuracy also differed significantly across prompts by Cochran’s Q test (Q(2) = 27.81, *p* < 0.001). Holm-adjusted exact pairwise McNemar tests showed significantly higher Full Accuracy for P3 than for P1 (*p* < 0.001) and P2 (*p* < 0.001), whereas P1 and P2 did not differ significantly (*p* = 0.625).

**Figure 4:**
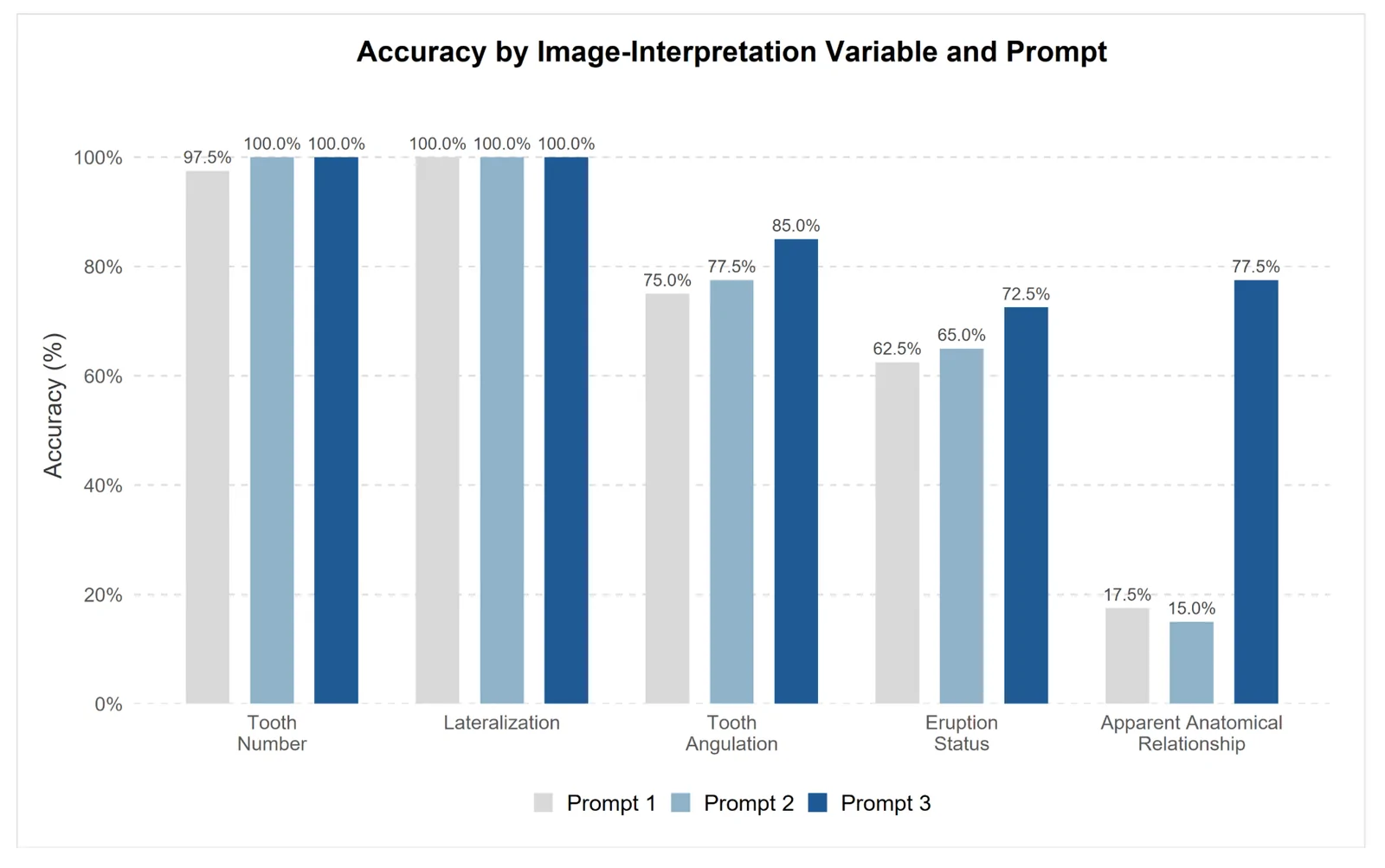
Image-interpretation variable accuracy across prompt architectures.

Image-interpretation variable accuracy and paired comparisons are presented in Table 3. Tooth Number accuracy was 97.5% in P1 and 100.0% in both P2 and P3, with no significant difference across prompt architectures after Holm adjustment. Lateralization was correctly identified in all cases across the three prompt architectures and was not statistically estimable because there was no within-image variation. Tooth Angulation accuracy was 75.0% in P1, 77.5% in P2, and 85.0% in P3, while Eruption Status accuracy was 62.5%, 65.0%, and 72.5%, respectively; neither variable differed significantly across prompt architectures after Holm adjustment. Apparent Anatomical Relationship showed the largest difference, with accuracy of 17.5% in P1, 15.0% in P2, and 77.5% in P3. This difference remained significant after Holm adjustment (Q = 38.77, Holm *p* < 0.001). Pairwise exact McNemar tests showed significant differences between P1 and P3 (*p* < 0.001) and between P2 and P3 (*p* < 0.001), whereas P1 and P2 did not differ significantly. The variable accuracy rates across prompt architectures are illustrated in Figure 4.

Error-pattern analyses further differentiated the prompting conditions (Table 4). The most frequent error patterns in P1 and P2 involved Apparent Anatomical Relationship, either as an isolated error (40.0% and 45.0%, respectively) or in combination with Eruption Status (25.0% in both conditions). Full Accuracy was observed in 4 of 40 reports (10.0%) for P1, 2 of 40 reports (5.0%) for P2, and 20 of 40 reports (50.0%) for P3. Reports with errors in three or more variables occurred in 12.5% of P1 reports and 7.5% of P2 reports, whereas no such error pattern was observed in P3. In P3, the remaining errors were distributed across isolated and two-variable error patterns, most frequently involving Eruption Status, Tooth Angulation, or Apparent Anatomical Relationship.

### AI Test–Retest Repeatability

AI test–retest repeatability was generally high across the three prompting conditions (Table 5). Lateralization showed complete agreement between Run 1 and Run 2 for P1, P2, and P3 (100%; AC1 = 1.000; κ = 1.000), while Tooth Number agreement ranged from 90.0% to 95.0%. Eruption Status agreement ranged from 87.5% to 92.5%. Tooth Angulation was less stable, with agreement of 87.5% for P1, 75.0% for P2, and 77.5% for P3. Apparent Anatomical Relationship showed high agreement for P1 (92.5%; AC1 = 0.920) and P2 (85.0%; AC1 = 0.833), but lower agreement for P3 (52.5%; AC1 = 0.433; κ = 0.271), representing the lowest repeatability observed.

Inter-rater agreement varied across the evaluated variables (Table 6). Complete agreement was observed for Tooth Number and Lateralization, with 100% agreement across all pairwise comparisons. For Tooth Angulation, observed agreement was 81.7% between Rater 1 and Rater 2, 84.2% between Rater 1 and the reference standard, and 77.5% between Rater 2 and the reference standard (Fleiss’ κ = 0.168; Gwet’s AC1 = 0.756). For Eruption Status, the corresponding agreement rates were 83.3%, 78.3%, and 66.7%, respectively (Fleiss’ κ = 0.140; Gwet’s AC1 = 0.669). A marked discrepancy was observed for Apparent Anatomical Relationship: agreement between the two independent raters was 85.0%, whereas agreement with the reference standard was 37.5% for both raters (Fleiss’ κ = −0.163; Gwet’s AC1 = 0.221). Full Accuracy showed the lowest agreement, with 60.8% agreement between the two raters, 45.8% between Rater 1 and the reference standard, and 30.0% between Rater 2 and the reference standard (Fleiss’ κ = −0.107; Gwet’s AC1 = −0.071).

## 4. Discussion

The performance of multimodal generative AI depends not only on the model itself, but also on how the task is framed and structured through prompts. A hierarchical structure allows prompts and intermediate decisions to be processed in sequential stages, while explicit gates instruct the model to verify each stage before proceeding. This principle supports the conceptual basis of the HRG framework examined in the present study.

The results support an advantage of the HRG architecture over the two simpler prompt conditions. Total Score was significantly higher with P3 than with both P1 and P2 (*p* < 0.001), whereas P1 and P2 did not differ significantly (*p* = 0.817). A similar pattern was observed for Full Accuracy, which was 10.0% with P1, 5.0% with P2, and 50.0% with P3. The proportion of reports with errors in three or more interpretation components was 12.5% with P1, 7.5% with P2, and 0% with P3. This pattern is consistent with the intended role of HRG in organizing linked image-analysis decisions through sequential validation. At the level of individual image-interpretation variables, only Apparent Anatomical Relationship differed significantly across prompt architectures after Holm adjustment. Accuracy for this variable was 17.5% for P1, 15.0% for P2, and 77.5% for P3. P3 performed significantly better than both P1 and P2. No significant differences were found for Tooth Number, Tooth Angulation, or Eruption Status. Lateralization was correctly identified in all cases across the three prompt architectures, and its omnibus test was not estimable because there was no within-image variation. The marked difference in Apparent Anatomical Relationship suggests that the effect of the HRG architecture was concentrated in a task requiring integration of spatial information rather than being uniformly distributed across all interpretation components. These findings are consistent with previous studies linking structured, sequential prompting to more stable reasoning and less ambiguous LLM outputs [20,21]. The gains observed in Apparent Anatomical Relationship may also reflect the anatomical reasoning difficulties previously described in LLM-based dental image analysis [20,22].

The higher performance observed with P3 supports the potential value of structuring complex tasks into sequential reasoning stages. In this context, Qasim et al. [14] reported that the RDoLT framework, which recursively decomposes complex reasoning tasks into structured subtasks and propagates information across reasoning stages, improved LLM performance across multiple text-based reasoning benchmarks. In our study, however, the difference between P2 and P3 extended beyond task decomposition and information propagation. P3 also incorporated additional verification procedures, more detailed instructions, and a longer prompt. Therefore, the present study cannot determine which individual feature was responsible for the observed performance improvement. The results should be interpreted as reflecting the effect of the complete P3 prompt as a whole, rather than as evidence that any single component independently produced the improvement.

These findings are compatible with the possibility that structured sequencing may influence the pattern of co-occurring errors. However, the present study did not directly observe causal error propagation. One possible explanation is that multimodal performance depends on the information included in a prompt as well as on the order in which that information is processed and controlled. By instructing the model to check each stage before proceeding, the framework was designed to reduce unsupported conclusions and potential errors. This strategy is consistent with prompt-engineering principles that emphasize sequential prompting, precise task specification, and logical reasoning [23]. Probing-based frameworks complement this approach by investigating how different layers of the model represent and process information [24]. The use of an orientation gate at an early stage is also supported by previous work on prompt programming, which suggests that early contextual guidance can improve the consistency of later reasoning steps [25]. A model may have the internal skills to process images and data, but this capability is useful only if the model produces accurate and consistent results when prompted [1,26].

To find a correct prompt instruction, instead of relying only on user intuition to construct a single prompt, an exploratory phase was used to identify recurrent failure types and iteratively refine and construct the prompts. This phase created an important preliminary step to guide the design of the three prompt architectures. P1 resembled a conventional zero-shot interaction, P2 incorporated five sequential steps followed by a final Internal Validation checkpoint, and P3 implemented the HRG framework through 10 macro-stages and 35 procedural control blocks, followed by a mandatory final audit.

The development of the proposed HRG framework followed the concept of human-centred generality proposed by Schellaert et al. [1]. The authors argue that the effectiveness of multimodal systems depends on the interaction among users, tasks, and prompting practices. This view also reflects the notion of AI systems as cognitive extenders, since model performance is shaped by how users organize and refine their interactions with the system. In this context, the HRG architecture translated recurrent failure patterns into explicit validation gates, while the experimental evaluation assessed accuracy and co-occurring error patterns across prompting conditions.

Qasim et al. [14] proposed RDoLT to improve LLM reasoning through hierarchical task decomposition and multi-feature evaluation of candidate thoughts. Their Knowledge Propagation Module (KPM) improves accuracy and reduces error propagation. HRG shares the principle that multimodal reasoning can be improved by breaking the task into steps and validating each step before moving to the next and before conclusions are formulated. Thus, HRG uses prompt architecture to structure the order of dependent analytical steps rather than merely guiding the final output.

In our study, the benchmark dataset provides a suitable testbed for hierarchical prompting because dental images require sequential decisions about localization, tooth identification, and anatomical relationships, and an error in an early decision may therefore affect later conclusions [14,18,27]. The apparent anatomical relationship between an impacted mandibular molar and the mandibular canal varies among individuals, reflecting a patient-specific anatomical configuration that cannot be reduced to a fixed template. This variability makes the task particularly demanding for MLLMs. Mogadala et al. [28] emphasized the importance of robust evaluation measures across different tasks and datasets. Their findings demonstrated that model performance can vary according to the dataset and evaluation context.

Several studies have evaluated MLLMs across clinical and healthcare applications. These evaluations have documented responses that appeared clinically plausible but contained inaccurate or fabricated information, such as invented surgical histories, fictitious symptoms [27,29], incorrect radiologic information [9], and nonexistent specialized medical equipment [12]. Although hierarchical gating has not yet been established as a standard framework for MLLMs, the literature suggests that performance can improve when prompts organize multimodal inference into defined stages, limit irrelevant context, and link low-level evidence to higher-level conclusions [30,31,32]. Previous studies in dental radiographic interpretation provide additional context for the influence of prompt formulation and task characteristics on MLLM performance. Suárez et al. [33] evaluated ChatGPT-4o for mandibular third-molar assessment using a short, standardized lay-language prompt and reported limited diagnostic accuracy together with only moderate reproducibility across repeated evaluations. Salmanpour and Akpınar [34], in contrast, used a chain-of-thought prompting strategy to assess the labiolingual position of impacted maxillary canines, but also reported limited performance, showing that the use of a structured prompting strategy did not eliminate performance limitations in that specific imaging task. These findings suggest that MLLM performance in dental image interpretation may depend not only on prompt design, but also on the anatomical target, task definition, and visual complexity.

Building on this context, the present study compared a short prompt, a structured sequential prompt, and a hierarchical prompt architecture incorporating explicit validation gates within the same dental imaging task. The performance observed in our experimental setting may also have been influenced by the manually delineated ROI, which may have directed model attention toward the target anatomy while preserving the full panoramic image. This possibility is consistent with Moncher et al. [35], who found that ROI selection can influence model performance in dental radiographic analysis.

To minimize unsupported clinical inferences, the three prompt conditions were evaluated using the same standardized experimental protocol. We used a retrospective observational dataset with expert radiology reference reports completed before model evaluation, thereby providing an independent evaluation standard, as detailed in our scoring methodology. The HRG framework was tested using a single MLLM and a controlled dental imaging benchmark to enable direct comparison among the prompt architectures while minimizing potential confounding factors.

Moreover, we assessed repeatability across two runs (Table 5) to determine whether the same case, evaluated using the same experimental protocol, would lead to the same structured clinical decision. Repeatability was high for two variables: Lateralization (100%) and Tooth Number (90.0–95.0%). In a systematic review, Maganur et al. [36] reported high performance of dental AI models for tooth detection and numbering, with precision values up to 99.4% and 98.0%, respectively. For Tooth Angulation, agreement varied from 75.0% to 87.5% across the three prompts. This variation may be related to the context-dependent nature of LLM inference, which relies on learned representations rather than task-specific geometric measurements [37]. The lowest repeatability in our study was observed for Apparent Anatomical Relationship for P3. This finding may be related to the difficulty of interpreting complex panoramic radiographs. Suárez et al. [33] reported that overlapping structures and underdeveloped roots were associated with incomplete or fabricated ChatGPT-4o responses, which may contribute to lower consistency across repeated evaluations.

Inter-rater agreement evaluated whether the two independent dental practitioners and the radiologist serving as the Reference Standard classified the AI outputs consistently. For Tooth Number, Lateralization, Tooth Angulation, and Eruption Status, pairwise observed agreement ranged from 66.7% to 100.0%. Apparent Anatomical Relationship was the main exception. Agreement between the two independent raters was 85.0%, but agreement with the Reference Standard was only 37.5% for each rater. This indicates that the disagreement occurred mainly in relation to the Reference Standard rather than between the two raters. This variability may reflect differences in how evaluators interpreted the anatomical relationship, as also reported in previous panoramic-radiography studies of mandibular third molars and the inferior alveolar canal [38,39]. Couto et al. [38] compared general dentists, oral surgeons, and oral radiologists and found that oral radiologists more frequently identified root dilacerations, retromolar canals, and probable contact between the roots and the mandibular canal. In a systematic review, Hegde et al. [39] identified clinical experience, knowledge, technical ability, and radiographic training as factors associated with interpretation errors. Full Accuracy also showed lower agreement, ranging from 30.0% to 60.8%. Because this outcome required all five variables to be classified consistently within the same evaluation, disagreement in any individual variable could affect the final Full Accuracy classification.

A further experimental control was the isolation of each inference in a separate Custom GPT conversation. According to OpenAI’s documentation, Custom GPTs do not use saved memory, custom instructions, or previous conversations, and each new conversation starts fresh [40]. Memory and personalization features were also disabled in the account settings as an additional precaution. After each inference, the chat was closed, and the next analysis was performed in a new conversation using one of the other two Custom GPTs. These procedures were adopted to minimize the possibility that information from a previous analysis could influence subsequent model outputs.

HRG provides a structured way to organize dependent decisions in multimodal dental image interpretation, with the complete architecture showing higher accuracy than the simpler prompt conditions.

### Limitations

The present proof-of-concept study has several limitations. The evaluation involved a single MLLM and a specific dental image-interpretation task, limiting the generalizability of the findings. Validation across additional models, imaging modalities, datasets, and clinical settings is therefore needed to assess the robustness and broader applicability of the observed performance differences.

The independent effect of ROI marking and its potential interaction with prompt type were not evaluated in the present study. Because the same ROI-marked panoramic image was used across all three prompt conditions, ROI marking was held constant and therefore did not confound the between-prompt comparison. However, the study was not designed to determine whether ROI marking altered model attention, task difficulty, or performance relative to workflows using unannotated panoramic images. These effects should be specifically evaluated in future studies comparing ROI-marked and unmarked images across different prompt architectures.

The experiment was conducted through the ChatGPT web interface rather than through an application programming interface. As a result, sampling parameters such as temperature, top-p, random seed, and token limits were not exposed and could not be manually controlled. Detailed internal model-version information and provider-side computational infrastructure were also unavailable. Although the same account, operating system, browser environment, and model option were used throughout the experiment, unobserved provider-side variation and random variation between model outputs cannot be excluded.

Another limitation is that the P3 prompt combined hierarchical sequencing, validation gates, checks, locks, a final audit, and more detailed domain-specific instructions within a longer prompt. Because these features were evaluated together and no content-matched non-gated control condition was included, the present study cannot determine whether the observed performance differences were attributable specifically to gating, prompt length, instruction content, or their combination. Future ablation studies using content-matched control conditions would help determine the individual contribution of these components. A limitation of the repeatability analysis concerns the coding of Apparent Anatomical Relationship. Some responses described projected proximity between the tooth and the mandibular canal. At the same time, they stated that three-dimensional contact or the buccolingual relationship could not be determined from panoramic radiography. In the adopted coding scheme, these responses could be classified as Not evaluable even when a projected relationship was described. This may have introduced classification bias. Future analyses should separate projected anatomical proximity from uncertainty about three-dimensional contact and report these categories independently.

The study also has a limitation regarding the reference standard, which was established by a single experienced oral and maxillofacial radiologist. Although this provided a consistent expert benchmark, future studies may benefit from consensus-based assessment involving multiple specialists.

The evaluation dataset included 40 panoramic radiographs. The limited sample size may reduce the precision of effect estimates and sensitivity to smaller differences between prompting conditions. Studies with larger independent samples are therefore warranted.

## 5. Conclusions

As a proof-of-concept investigation, this study evaluated the Hierarchical Reasoning Gates (HRG) framework as a prompt-level architecture designed to control inferential dependencies in multimodal large language models. The HRG architecture was associated with improved image-interpretation performance and fewer complex error patterns than the simpler prompting approaches. These findings support the potential value of structured prompt architectures for guiding multimodal reasoning, while emphasizing that performance and repeatability may vary across specific interpretation tasks. Expert supervision remains essential for clinical applications.

## Figures and Tables

**Table 1:** Descriptive statistics for Total Score across prompt architectures.

Prompt Architecture	*n*	Mean ± SD	Median (Q1–Q3)	Minimum–Maximum
P1	40	3.52 ± 0.85	4.0 (3.0–4.0)	2–5
P2	40	3.58 ± 0.71	4.0 (3.0–4.0)	2–5
P3	40	4.35 ± 0.74	4.5 (4.0–5.0)	3–5

Note: SD, standard deviation; Q1, first quartile; Q3, third quartile. Total Score ranged from 0 to 5.

**Table 2:** Overall paired performance analysis across prompt architectures.

Outcome	Overall Test	Statistic	Overall *p*-Value	Effect Size	Holm-Adjusted Pairwise *p*-Values
Total Score	Friedman test	$\chi^{2}$ = 36.873	<0.001	Kendall’s W = 0.461	P1 vs. P2 = 0.817
					P1 vs. P3 < 0.001
					P2 vs. P3 < 0.001
Full Accuracy	Cochran’s Q test	Q = 27.81	<0.001	—	P1 vs. P2 = 0.625
					P1 vs. P3 < 0.001
					P2 vs. P3 < 0.001

Note: Total Score was analyzed using the Friedman test followed by pairwise Wilcoxon signed-rank tests with Holm adjustment. Full Accuracy was analyzed using Cochran’s Q test followed by exact McNemar pairwise comparisons with Holm adjustment.

**Table 3:** Accuracy and statistical comparison of image-interpretation variables across prompt architectures.

Variables	P1, *n* (%)	P2, *n* (%)	P3, *n* (%)	Q	Holm *p*	Pairwise Comparisons
Tooth Number	39/40 (97.5%)	40/40 (100.0%)	40/40 (100.0%)	2.00	0.788	—
Lateralization	40/40 (100.0%)	40/40 (100.0%)	40/40 (100.0%)	—	—	—
Tooth Angulation	30/40 (75.0%)	31/40 (77.5%)	34/40 (85.0%)	2.89	0.788	—
Eruption Status	25/40 (62.5%)	26/40 (65.0%)	29/40 (72.5%)	3.25	0.788	—
Apparent Anatomical Relationship	7/40 (17.5%)	6/40 (15.0%)	31/40 (77.5%)	38.77	<0.001	P1 vs. P2 = 1.000
						P1 vs. P3 < 0.001 *
						P2 vs. P3 < 0.001 *

Q = Cochran’s Q statistic. Holm *p* = Cochran’s Q *p*-value adjusted across the five variables. Pairwise *p*-values correspond to exact McNemar tests with Holm adjustment. Pairwise comparisons were performed only when the Holm-adjusted omnibus test was significant. — indicates not estimable for Q/Holm *p* or not performed for pairwise comparisons. * *p* < 0.05.

**Table 4:** Distribution of errors across prompt conditions (*n* = 40).

Error Pattern	Prompt 1, *n* (%)	Prompt 2, *n* (%)	Prompt 3, *n* (%)
Full Accuracy (Error-free)	4 (10.0%)	2 (5.0%)	20 (50.0%)
Apparent Anatomical Relationship only	16 (40.0%)	18 (45.0%)	4 (10.0%)
Tooth Angulation only	2 (5.0%)	3 (7.5%)	4 (10.0%)
Tooth Number only	0 (0.0%)	0 (0.0%)	0 (0.0%)
Eruption Status only	0 (0.0%)	1 (2.5%)	6 (15.0%)
Tooth Number + Apparent Anatomical Relationship only	0 (0.0%)	0 (0.0%)	0 (0.0%)
Tooth Angulation + Apparent Anatomical Relationship only	2 (5.0%)	3 (7.5%)	1 (2.5%)
Tooth Angulation + Eruption Status only	0 (0.0%)	0 (0.0%)	1 (2.5%)
Tooth Number + Tooth Angulation only	1 (2.5%)	0 (0.0%)	0 (0.0%)
Eruption Status + Apparent Anatomical Relationship only	10 (25.0%)	10 (25.0%)	4 (10.0%)
≥3 variable errors	5 (12.5%)	3 (7.5%)	0 (0.0%)

Note: Error patterns were classified according to the combination of incorrect image-interpretation variables within each report. Reports with three or more simultaneous errors were grouped as ≥3 variable errors. Full Accuracy denotes an error-free report. Overall, P3 showed higher Total Scores and a greater proportion of error-free reports than P1 and P2, whereas no significant difference was observed between P1 and P2 in the corresponding pairwise comparisons. Reports with errors in three or more variables were observed in P1 and P2 but not in P3.

**Table 5:** AI test–retest repeatability of structured classifications across prompting conditions.

Prompt	Variable	Observed Agreement (Run 1 vs. Run 2), *n* (%)	Observed Agreement, 95% CI	Gwet’s AC1 (95% CI)	Cohen’s κ
P1	Tooth Number	36 (90.0%)	76.9–96.0	0.864 (0.721–0.968)	0.810
P1	Lateralization	40 (100.0%)	91.2–100.0	1.000 (1.000–1.000)	1.000
P1	Tooth Angulation	35 (87.5%)	73.9–94.5	0.841 (0.706–0.968)	0.804
P1	Eruption Status	37 (92.5%)	80.1–97.4	0.919 (0.805–1.000)	0.469
P1	Apparent Anatomical Relationship	37 (92.5%)	80.1–97.4	0.920 (0.809–1.000)	0.375
P2	Tooth Number	38 (95.0%)	83.5–98.6	0.939 (0.844–1.000)	0.909
P2	Lateralization	40 (100.0%)	91.2–100.0	1.000 (1.000–1.000)	1.000
P2	Tooth Angulation	30 (75.0%)	59.8–85.8	0.683 (0.503–0.843)	0.608
P2	Eruption Status	35 (87.5%)	73.9–94.5	0.867 (0.750–0.974)	0.461
P2	Apparent Anatomical Relationship	34 (85.0%)	70.9–92.9	0.833 (0.678–0.948)	0.259
P3	Tooth Number	36 (90.0%)	76.9–96.0	0.879 (0.753–0.971)	0.811
P3	Lateralization	40 (100.0%)	91.2–100.0	1.000 (1.000–1.000)	1.000
P3	Tooth Angulation	31 (77.5%)	62.5–87.7	0.739 (0.589–0.884)	0.673
P3	Eruption Status	36 (90.0%)	76.9–96.0	0.884 (0.761–0.972)	0.765
P3	Apparent Anatomical Relationship	21 (52.5%)	37.5–67.1	0.433 (0.248–0.618)	0.271

Note: *n*, cases with identical classifications between Run 1 and Run 2; CI, confidence interval.

**Table 6:** Inter-rater agreement between the two independent raters and the Reference Standard (RS).

Variable	Rater 1 vs. Rater 2, %	Rater 1 vs. RS, %	Rater 2 vs. RS, %	Fleiss’ κ	Gwet’s AC1
Tooth Number	100.0	100.0	100.0	1.000	1.000
Lateralization	100.0	100.0	100.0	NE	1.000
Tooth Angulation	81.7	84.2	77.5	0.168	0.756
Eruption Status	83.3	78.3	66.7	0.140	0.669
Apparent Anatomical Relationship	85.0	37.5	37.5	−0.163	0.221
Full Accuracy	60.8	45.8	30.0	−0.107	−0.071

Note: Pairwise values represent observed agreement. N = 120 image–prompt evaluations per variable. NE, not estimable.

## Data Availability

The clinical images belong to an internal Dental Polyclinic of the State University of Amazonas (UEA) institutional repository and are not publicly distributed under a public license. Access is subject to institutional data-confidentiality and data-protection requirements. The dataset source, selection criteria, ROI preparation, reference standard, and experimental workflow are described in the article; the complete prompt architectures and fixed task command are provided in Supplementary Material S1.

## Supplementary Materials

The following supporting information can be downloaded at: https://www.mdpi.com/article/10.3390/jimaging12100508/s1, Supplementary Material S1: Complete Verbatim Prompts and Fixed Task Command; complete operational structure and verbatim wording of P1, P2, and P3, including the fixed task command used for every inference.

## Abbreviations

The following abbreviations are used in this manuscript:

AC1	Gwet’s Agreement Coefficient 1
AI	Artificial Intelligence
CBCT	Cone-Beam Computed Tomography
CoT	Chain-of-Thought
CUI	Conversational User Interface
FDI	Fédération Dentaire Internationale
GPT	Generative Pre-trained Transformer
HRG	Hierarchical Reasoning Gates
Inserm	National Institute of Health and Medical Research
InTheRNA	Innovative Therapies and Nanomedicine
KPM	Knowledge Propagation Module
LGPD	Brazilian General Data Protection Law
LLM	Large Language Model
MLLM	Multimodal Large Language Model
P1	Prompt Architecture 1 (baseline)
P2	Prompt Architecture 2 (structured)
P3	Prompt Architecture 3 (Hierarchical Reasoning Gates)
PNG	Portable Network Graphics
Q1	First Quartile
Q3	Third Quartile
RDoLT	Recursive Decomposition of Logical Thoughts
ROI	Region of Interest
RS	Reference Standard
SD	Standard Deviation
UEA	State University of Amazonas
